# Transcription Factor-Based Fate Specification and Forward Programming for Neural Regeneration

**DOI:** 10.3389/fncel.2020.00121

**Published:** 2020-05-20

**Authors:** Lea J. Flitsch, Karen E. Laupman, Oliver Brüstle

**Affiliations:** Institute of Reconstructive Neurobiology, Life & Brain Center, University of Bonn Medical Faculty and University Hospital Bonn, Bonn, Germany

**Keywords:** forward programming, transcription factor-driven differentiation, direct cell fate conversion, biomedical application, translation, transplantation, brain repair

## Abstract

Traditionally, *in vitro* generation of donor cells for brain repair has been dominated by the application of extrinsic growth factors and morphogens. Recent advances in cell engineering strategies such as reprogramming of somatic cells into induced pluripotent stem cells and direct cell fate conversion have impressively demonstrated the feasibility to manipulate cell identities by the overexpression of cell fate-determining transcription factors. These strategies are now increasingly implemented for transcription factor-guided differentiation of neural precursors and forward programming of pluripotent stem cells toward specific neural subtypes. This review covers major achievements, pros and cons, as well as future prospects of transcription factor-based cell fate specification and the applicability of these approaches for the generation of donor cells for brain repair.

## Introduction

Identifying treatment options for neurological and especially neurodegenerative diseases is one of the most pressing tasks of modern biomedicine. In this context, neural cell replacement has emerged as a particularly promising strategy, which has gained further impetus with the availability of massively scalable human PSCs. A key prerequisite for the use of human PSCs in neural repair is the efficient derivation of disease-specific cell populations. Over the last 20 years, numerous *in vitro* differentiation protocols were established. Classically, they involve extrinsic factors such as morphogens to guide the differentiation process toward a specific cell fate, thereby mimicking regionalization processes during nervous system development. This approach has led to significant advances, for instance, for the generation of midbrain dopamine neurons for the treatment of PD (Kriks et al., 2011; Kirkeby et al., 2012). However, the generation of many neural subtypes is frequently complicated by long differentiation times and complex multi-step growth factor-regimens, which often yield cultures exhibiting a high degree of heterogeneity (see also review by Tao and Zhang, 2016). Thus, many growth factor-based protocols have to be regarded as insufficiently precise when it comes to fine-tuning the specification of distinct neural subtypes, especially considering future biomedical applications.

Since morphogen-based cell specification finally converges on the activation of specific transcriptional programs, TF overexpression by itself represents an alternative method to guide cell fate acquisition. This idea was further fueled by the ground-breaking discovery by Takahashi and Yamanaka that an ESC-like pluripotent fate can be induced in mouse (Takahashi and Yamanaka, 2006) and human (Takahashi et al., 2007) somatic cells by overexpressing a combination of four different TFs, namely Oct3/4, Sox2, Klf4 and c-Myc. The introduction of the iPSC reprogramming technology had two major implications for the scientific field: First, the feasibility to reprogram terminally differentiated somatic cells into iPSCs hinted at the potential power of exploiting TF overexpression as a tool to manipulate cell fates more globally. Second, it created the general opportunity to derive neural cells from basically any adult human and thus revealed new avenues for disease modeling and personalized biomedicine.

In line with the first idea is the concept of direct cell fate conversion, i.e., the use of TFs to directly convert one somatic cell type into another without transiting a stable, pluripotent state. In fact, direct cell fate conversion has been achieved far before the iPSC technique was even introduced: Davis et al. (1987) successfully converted mouse fibroblasts into myoblasts by overexpressing the TF Myod3. As for neurons, it had already been shown by Magdalena Götz and colleagues in the early 2000s that mouse astrocytes can be directly converted into neurons by overexpressing single neural TFs such as Pax6 (Heins et al., 2002), Olig2 (Buffo et al., 2005), Ngn2 and Ascl1 (Berninger et al., 2007). In 2010, the Wernig lab achieved to derive iNs from mouse fibroblasts via transdifferentiation across germ layers (Vierbuchen et al., 2010). Although in this case Ascl1 overexpression seemed sufficient to drive neuronal conversion, too, the derivation of mature iNs was most efficient when multiple TFs were used simultaneously, such as the combined expression of Ascl1, Brn2 and Myt1l (Vierbuchen et al., 2010). This TF cocktail alone (Pfisterer et al., 2011a, b) or in combination with the bHLH TF NEUROD1 (Pang et al., 2011) was shown to suffice for inducing iNs from human fibroblasts. In combination with SOX2, ASCL1 can also convert human non-neural, brain-resident pericytes into functional iNs (Karow et al., 2012, 2018). How broadly TF overexpression can impact the differentiation of PSCs is illustrated by studies of Minoru Ko and colleagues, who established more than 180 mouse ESC lines, each expressing a distinct TF from the *ROSA* locus after doxycycline induction, which resulted in the specification of a large variety of different somatic cell lineages (in the following also referred to as ‘forward programming’; Nishiyama et al., 2009; Correa-Cerro et al., 2011; Yamamizu et al., 2016).

**FIGURE 1:**
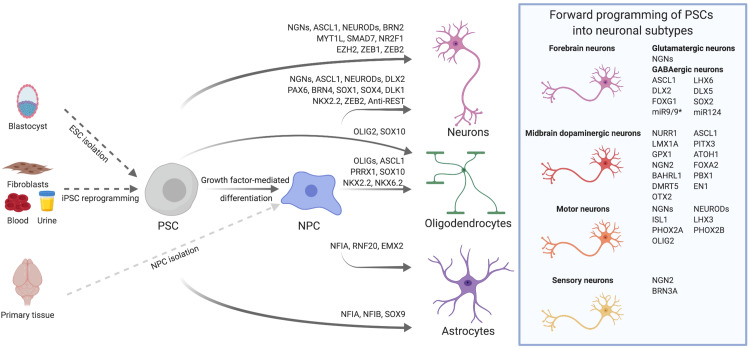
Transcription factor-mediated specification of pluripotent stem cells and neural precursor cells. PSCs, such as ESCs derived from the blastocyst or iPSCs reprogrammed from somatic cells, as well as primary or PSC-derived NPCs can be differentiated into neurons, astrocytes and oligodendrocytes via overexpression of cell type-specific TFs. By using subtype- and/or region-specific TFs, forward programming approaches can be further refined to yield highly specified neuronal subtypes as for instance midbrain dopaminergic neurons. The TFs listed for the derivation of diverse neural cell types were either used alone or in combination. Further details on TF combinations are provided in the main text and in Tables 1, 2.

The aim of this review is to give a comprehensive overview on TF-based approaches for the generation of neural cells (Figure 1). We will speculate on general mechanisms underlying TF-mediated neuronal differentiation and forward programming, specifically comment on current efforts to derive clinically relevant neuronal subtypes and glial cells, and summarize recent endeavors to apply these cells *in vivo* for brain repair. Finally, we will discuss forward programming as an alternative to direct cell fate conversion, and comment on the achievements as well as remaining hurdles for biomedical translation.

## Derivation of Neural Cell Types Via Forward Programming

Specifying cell fates by TF overexpression is comparably easy to accomplish within one lineage, especially when starting from cell types which are direct progenitors of the target cell type. Almost 20 years ago, Sun et al. (2001) reported about the successful derivation of neurons by retrovirally overexpressing the pro-neural bHLH TF Ngn1 in primary rat cortical NPCs (Table 1). Since then, other TFs belonging to the bHLH family have been shown to be capable of forcing neuronal differentiation from different NPC populations. These TFs include various Ngns such as Ngn1 (Serre et al., 2012; Song et al., 2017), Ngn2 (Geoffroy et al., 2009; Serre et al., 2012; Bolós et al., 2014; Ho et al., 2016; Li X. et al., 2016) and Ngn3 (Serre et al., 2012), Ascl1 (Geoffroy et al., 2009; Kim et al., 2009; Serre et al., 2012; Li X. et al., 2016; Barretto et al., 2020) as well as Neurod TFs (Hsieh et al., 2004).

**TABLE 1:** Transcription factors used for promoting neuronal differentiation of neural precursor cells and pluripotent stem cells *in vitro*.

Desired cell type	Starting cell type	Species	Transcription factor used for forward programming	References
Neurons (generic)	NPCs	Mouse	Pax6	Hack et al. (2004)
	NPCs	Mouse	Sox1	Kan et al. (2004)
	NPCs	Mouse	Dominant-negative form of REST	Greenway et al. (2007)
	NPCs	Mouse	Ascl1 or Ngn2	Geoffroy et al. (2009)
	NPCs	Mouse	Ngn2	Bolós et al. (2014)
	NPCs	Mouse	Sox4	Braccioli et al. (2018)
	NPCs	Mouse	Zeb2	Yang et al. (2018)
	NPCs	Rat	Ngn1	Sun et al. (2001)
	NPCs	Rat	Neurod	Hsieh et al. (2004)
	NPCs	Rat	Brn4	Shi et al. (2010)
	NPCs	Rat	Brn4	Tan et al. (2010)
	NPCs (under glia-promoting conditions)	Rat	Dominant-negative form of REST	DeWald et al. (2011)
	ESC-derived NPCs	Mouse and human	Dlk1	Surmacz et al. (2012)
	NPCs	Human	ASCL1	Kim et al. (2009)
	NPCs	Human	ASCL1 or NGN1 or NGN2 or NGN3	Serre et al. (2012)
	NPCs	Human	ASCL1 or NGN2 or ASCL1+NGN2	^(1)^ Li X. et al. (2016)
	NPCs (primary and ESC-derived)	Human	shREST or shHAUSP	Huang et al. (2011)
	iPSC-derived NPCs	Human	NGN2	Ho et al. (2016)
	iPSC-derived NPCs	Human	ASCL1+DLX2	Barretto et al. (2020)
	ESCs	Mouse	Neurod1 or Neurod2 or Neurod3	O’Shea (2001)
	ESCs	Mouse	Ngn1	Tong et al. (2010)
	ESCs	Mouse	Ngn2	Thoma et al. (2012)
	ESCs	Mouse	Ascl1 or Smad7 or Nr2f1	Yamamizu et al. (2013)
	ESCs	Mouse	Multiple (TF screen)	Teratani-Ota et al. (2016)
	ESCs	Mouse	Neurod1	Pataskar et al. (2016)
	ESCs	Mouse	Multiple (TF screen)	Liu et al. (2018)
	ESCs	Mouse	sh-lnc-RNA-1604+Zeb1 and/or Zeb2	Weng et al. (2018)
	ESCs	Mouse	Ngn2 or Ascl1	Aydin et al. (2019)
	PSCs	Human	ASCL1+ BRN2+MYT1L	Pang et al. (2011)
	PSCs	Human	NGN2 or NEUROD1	^(3)^ Zhang et al. (2013)
	ESCs	Human	ASCL1	Chanda et al. (2014)
	iPSCs	Human	FOXG1+SOX2+ASCL1+DLX5+LHX6	Colasante et al. (2015)
	PSCs	Human	ASCL1+DLX2+LHX6+miR9/9*-124	^(6)^ Sun et al. (2016)
	iPSCs	Human	ASCL1	Robinson et al. (2016)
	iPSCs	Human	NGN2	Rubio et al. (2016)
	PSCs	Human	ASCL1+DLX2	^(4)^ Yang et al. (2017)
	iPSCs	Human	NGN2	Frega et al. (2017)
	PSCs	Human	NGN1 or NGN2 or NGN3 or NEUROD1 or NEUROD2	Goparaju et al. (2017)
	ESCs	Human	NGN1 or NGN2 or NGN3 or NEUROD1 or NEUROD2	Matsushita et al. (2017)
	PSCs	Human	NGN2	Pawlowski et al. (2017)
	iPSCs	Human	NGN2	Wang et al. (2017)
	PSCs	Human	NGN2	Nehme et al. (2018)
	PSCs	Human	LHX6	^(5)^ Yuan et al. (2018)
	ESCs	Human	ZEB1	^(2)^ Jiang et al. (2018)
	iPSCs	Human	NGN2	Meijer et al. (2019)
	iPSCs	Human	NGN2 or ASCL1+DLX2	Rhee et al. (2019)
	iPSCs	Human	NGN2	Nickolls et al. (2020)
Dopaminergic neurons	NPCs	Mouse	Nurr1	Soldati et al. (2012)
	NPCs	Mouse	Foxa2	Kittappa et al. (2007)
	NPCs	Rat	Nurr1	Sakurada et al. (1999)
	NPCs	Rat	Nurr1	^(10)^ Kim et al. (2003)
	NPCs	Rat	Nurr1 or Ascl1+Nurr1 or Nurr1+Ngn1 or Nurr1+Ngn2 or Nurr1+Shh or Nurr1+Bcl-XL or Nurr1+Bcl-XL+Shh	^(11)^ Park et al. (2006)
	NPCs	Rat	Nurr1	Bae et al. (2009)
	ESC-derived NPCs	Mouse	Dmrt5	Gennet et al. (2011)
	ESC-derived NPCs	Mouse	Lmx1a	Andersson et al. (2006)
	ESC-derived NPCs	Mouse	Lmx1a or En1 or Otx2 or Lmx1a+En1 or Lmx1a+Otx2 or Lmx1a+En1+Otx2 or Lmx1a+En1+Otx2+Foxa2	Panman et al. (2011)
	ESC-derived NPCs	Mouse	Lmx1a+Foxa2 or Lmx1a+Foxa2+Barhl1	Kee et al. (2017)
	PSC-derived NPCs	Human	PBX1	Villaescusa et al. (2016)
	ESC-derived NPCs	Human	ASCL1, FOXA2, LMX1A, NGN2, NURR1, OTX2 and PITX3 (alone or in combination)	Azimi et al. (2018)
	ESC-derived NPCs	Human	LMX1A	^(12)^ Friling et al. (2009)
	ESCs	Mouse	Nurr1	Chung et al. (2002)
	ESCs	Mouse	Nurr1	^(8)^ Kim et al. (2002)
	ESCs	Mouse	Gpx1+Nurr1	Abasi et al. (2012)
	iPSCs	Mouse	Nurr1+Pitx3	Salemi et al. (2016)
	ESCs	Mouse and human	Nurr1+Pitx3	^(9)^ Martinat et al. (2006)
	PSCs	Human	LMX1A	^(13)^ Sánchez-Danés et al. (2012)
	iPSCs	Human	mAscl1+mNurr1+mLmx1a	^(7)^ Theka et al. (2013)
	PSCs	Human	ATOH1	Sagal et al. (2014)
	iPSCs	Human	NGN2 and/or ATOH1	Xue et al. (2019)
Medium spiny neurons	ESC-derived NPCs	Human	GSX2+EBF1	^(14)^ Faedo et al. (2017)
Motor neurons	ESC-derived NPCs	Mouse	Phox2b or Olig2	Panman et al. (2011)
	ESC-derived NPCs	Mouse and human	Phox2a or Phox2b	Mong et al. (2013)
	ESCs-derived NPCs	Human	NGN2+ISL1+LHX3	Hester et al. (2011)
	ESCs	Mouse	Ngn2+Isl1+Lhx3 or Ngn2+Isl1+Phox2a	^(15)^ Mazzoni et al. (2013)
	iPSCs	Human	NGN1+NGN2	Busskamp et al. (2014)
	PSCs	Human	NGN1+NGN2+NGN3+NEUROD1+NEUROD2	Goparaju et al. (2017)
	PSCs	Human	NGN2+ISL1+LHX3	Goto et al. (2017)
	iPSCs	Human	NGN2+ISL1+LHX3 or NGN2+ISL1+PHOX2A	De Santis et al. (2018)
Sensory neurons	ESC-derived neural crest progenitors	Human	NGN2	Schrenk-Siemens et al. (2015)
	iPSC-derived neural crest progenitors + iPSCs	Human	NGN2+BRN3A	Nickolls et al. (2020)
Otic neurons	(Otic) NPCs	Human	NGN1	Song et al. (2017)
Serotonergic neurons	ESC-derived NPCs	Mouse	Nkx2.2	Panman et al. (2011)

**FIGURE 2:**
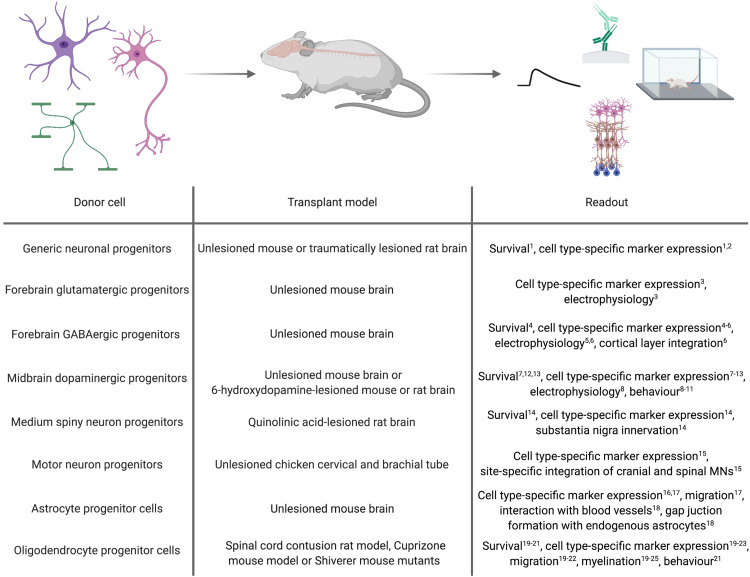
*In vivo* applications of cell types derived by forward programming. Numbers in superscript relate to references cited in Tables 1, 2.

*Numbers in superscript relate to citations in Figure 2.*

### bHLH Transcription Factors as Key Instructors of Neuronal Differentiation

Interestingly, also rapid neuronal differentiation of PSCs (this term will be used to describe ESCs and iPSCs together in the following), which are not yet committed to the neural lineage, was shown to be feasible with bHLH TFs. A milestone in the field of neuronal forward programming was reached in 2011, when the groups of Marius Wernig and Thomas Südhof reported that combined overexpression of the TFs Ascl1, Brn2, and Myt1l not only suffices to transdifferentiate mouse fibroblasts into neurons (Vierbuchen et al., 2010) but also efficiently drives neuronal specification from human PSCs (Pang et al., 2011). The authors revealed that ASCL1 is most crucial for neural fate acquisition, whereas the TFs BRN2 and MYT1L rather promote down-stream neuronal maturation. Using the full TF cocktail, electrophysiologically active neurons can be derived from human PSCs after only 6 days of *in vitro* differentiation (Pang et al., 2011). Several other labs subsequently demonstrated that Ascl1 alone can efficiently forward program mouse ESCs (Yamamizu et al., 2013; Teratani-Ota et al., 2016) and human PSCs (Chanda et al., 2014; Robinson et al., 2016) toward a neuronal fate, albeit with slower differentiation dynamics than the full ASCL1, BRN2, and MYT1L TF combination: In mouse ESCs, overexpression of Ascl1 leads to a sharp increase of neural markers within the first 7 days of induction (Yamamizu et al., 2013; Teratani-Ota et al., 2016), and 11 days after overexpressing Ascl1, about half of the neurons were shown to generate action potentials upon current injection (Yamamizu et al., 2013). In human PSCs, ASCL1-overexpressing cells do not start to express neuronal markers such as TUBB3 and MAP2 before day 9 of differentiation. Still, ASCL1 overexpression alone is sufficient to generate morphologically and functionally mature neurons when ESC-derived immature neuronal cells are cultured in advanced neuronal differentiation medium and are grown on primary glial cells. These neurons not only exhibit mature electrophysiological properties such as spontaneous action potential firing after 4 weeks of differentiation but also respond to exogenous AMPA and GABA application and demonstrate signs of short-term synaptic plasticity, indicating the formation of functional synapses (Chanda et al., 2014).

Overexpression of other bHLH TFs, too, induces rapid neuronal differentiation of PSCs: In one of the first *in vitro* studies employing overexpression of Ngn1 in mouse ESCs, transduced cells underwent morphological rearrangements forming neurite-like structures already within the first 72 h and became electrophysiologically excitable as early as 4 days after transgene induction (Tong et al., 2010). After 5 days of Ngn2 overexpression, mouse ESC-derived cells express the mature neuronal marker Map2, display neuronal electrophysiological properties at day 10, and form synapses in co-culture with primary mouse hippocampal neurons 20 days post induction (Thoma et al., 2012). The first ground-breaking proof that NGN2 has the same effect in human PSCs was – again – provided by the groups of Marius Wernig and Thomas Südhof in 2013. The authors demonstrated that forward programming human PSCs with NGN2 reproducibly yields neurons with almost 100% purity within 2 weeks, and as was observed in mouse cells, these neurons do not only acquire neuronal-like electrophysiological properties but are also capable of functionally integrating into synaptic networks with cortical mouse neurons. Notably, the authors further reported that overexpressing the bHLH TF NEUROD1 can instruct neuronal differentiation from human PSCs, too (Zhang et al., 2013). Interestingly, already in 2001, O’Shea (2001) had investigated the neurogenic effect of Neurod TFs by overexpressing Neurod1, Neurod2, and Neurod3 in mouse ESCs and found that all three Neurods suffice to induce immature neuronal-like cells within 72 h.

More recently, other groups have corroborated the finding that NGN2 overexpression suffices to forward program human PSCs to neurons expressing MAP2 and NEUN within 2 to 10 days of differentiation (Rubio et al., 2016; Goparaju et al., 2017; Matsushita et al., 2017; Pawlowski et al., 2017). Neuronal differentiation can be further accelerated by combined overexpression of NGN1 and NGN2, and in this case, 90% of all cells were found to express MAP2 and the synapse marker synapsin already at day 4 of differentiation. However, additional morphological, transcriptomic, and functional analyses indicate that a majority of the obtained neurons at this early time point are still immature and not yet fully developed (Busskamp et al., 2014). Yet, several labs including our own have revealed that co-culturing NGN2-neurons with glial cells significantly facilitates maturation (Zhang et al., 2013; Busskamp et al., 2014; Meijer et al., 2019; Rhee et al., 2019), and when cultured under appropriate conditions, neurons derived by forward programming can be utilized for sophisticated electrophysical analyses: Cultured on glia microdots, single forward programmed NGN2-neurons form an autaptic system by making synapses onto themselves, which can be used for studying functional features such as synaptic transmission and short-term plasticity (Meijer et al., 2019; Rhee et al., 2019), and networks of forward programmed neurons cultured on multielectrode arrays have also been employed for functional analyses (Frega et al., 2017; Nehme et al., 2018).

### Mechanisms Underlying bHLH Transcription Factor-Mediated Forward Programming

bHLH TFs are named after their common protein structure motif consisting of two α-helices mediating dimerization and a basic domain, which binds to E-box motifs with the consensus sequence CANNTG. The group of bHLH TFs is subdivided according to their ubiquitous versus tissue-specific expression profile, and neural bHLH TFs are further grouped into the *achaete-scute complex* and *atonal* gene families (for further details see review by Dennis et al., 2019). First hints as of why bHLH TFs might be able to orchestrate neuronal fate acquisition were obtained from NPC-to-neuron differentiation paradigms: Ngn1, for instance, specifically binds to E-box motifs at neuronal genes in rat NPCs, acting as a direct transcriptional activator (Sun et al., 2001). In human PSCs, the TFs NGN1, NGN2 and NGN3 seem to even cross-activate each other and induce common pro-neural down-stream targets including other bHLH TFs such as *NEUROD1, NEUROD2* and *NEUROD4* (Busskamp et al., 2014; Goparaju et al., 2017; Xue et al., 2019). Such a synergism might not be restricted to the group of NGNs, since the bHLH TF ATOH1 has been shown to induce both, *NGN2* and *NEUROD1* in human PSCs, and was thus used for forward programming of human PSCs into neurons (Sagal et al., 2014; Xue et al., 2019). These observations might indicate that one common mechanism underlying neuronal forward programming of PSCs with bHLH TFs is the activation of a whole network of cross-regulated bHLH TFs, including the induction of more down-stream Neurod TFs. In line with this hypothesis, no difference was reported comparing the neuronal induction potency of NGN1, NGN2, NGN3, NEUROD1, and NEUROD2 in human ESCs (Matsushita et al., 2017).

In order to elucidate the mechanism by which combined NGN1 and NGN2 overexpression drives neuronal fate acquisition of human iPSCs more in depth, Busskamp et al. (2014) performed a comprehensive set of experiments dissecting mRNA and miRNA regulation kinetics during the early phase of neural induction. A mRNA network analysis revealed that during the first 4 days of differentiation, destabilization of the pluripotency network is initiated by decreasing *SOX2, NANOG*, and *OCT4* levels, and genes associated with an NPC stage and the gene ontology term ‘regulation of neurogenesis’ such as *NOTCH1*, *DLL1*, *DLL4*, *HES5*, *FABP7*, and *NTN1* are temporarily upregulated. This phase of NPC marker induction is very brief, though (as was also observed by Zhang et al., 2013), resulting in a significant downregulation of cell cycle-related genes by day 4, which suggests that PSC-derived cells only traverse a short progenitor-like phase during forward programming. In line with this, neuron-associated genes such as *POU3F2* (also known as *BRN2*), *ZEB1*, *ISL1*, *TLX3*, and *POU4F1* (also known as *BRN3a*) are upregulated already in this early phase of reprogramming, whilst inhibitors of neurogenesis as for instance *REST* and *HES1* are repressed. Concomitant with the dynamics of mRNA regulation, the expression of the pluripotency-associated miRNA cluster 302/367 decreases, whereas the abundance of neuronal miRNAs such as miR124, miR96, and miR9 increases upon differentiation (Busskamp et al., 2014).

The molecular consequences of Neurod1 induction were explicitly investigated by Vijay Tiwari’s group in mouse ESCs: Neurod1 overexpression influences chromatin accessibility at its target sites by reducing repressive H3K27me3 marks, increasing H3K27ac and recruiting RNA polymerase II. Hence, during the first 24 h of differentiation, the fraction of enhancer and promotor regions directly bound by Neurod1 probably accounts for approximately 25% of all upregulated genes identified by RNA sequencing. Interestingly, upregulated genes are exclusively enriched for neurogenesis-associated gene ontology terms, and consequently, after 48 h, and even in the presence of the pluripotency-promoting factor Lif, Tubb3 expression is induced. Even transient Neurod1 expression for as short as 48 h suffices to stably remodel the epigenetic and transcriptional landscape at Neurod1 targets, which later drive neuronal differentiation of ESCs even in the absence of Neurod1 (Pataskar et al., 2016). Altogether, these data indicate that activation of the bHLH TF Neurod1 might represent one of the crucial entry points for neural fate acquisition in forward programming paradigms.

### Neuronal Forward Programming Factors Beyond the bHLH Family

Neuronal specification from diverse NPC populations can be driven by pro-neural, non-bHLH TFs such as Pax6 (Hack et al., 2004), Brn4 (Shi et al., 2010; Tan et al., 2010) and members of the Sox family of TFs (Kan et al., 2004; Braccioli et al., 2018). Furthermore, overexpression of Dlk1 in mouse and human ESC-derived NPCs facilitates neural specification by promoting cell cycle exit via reduction of Notch, and modulation of BMP signaling (Surmacz et al., 2012). Finally, decreasing REST signaling in human NPC lines induces neurogenesis, too (Huang et al., 2011), and this can most likely be attributed to REST’s function as a transcriptional repressor of neuronal genes such as *Tubb3* (Greenway et al., 2007) and *Neurod2* (DeWald et al., 2011).

Considering these observations, it is not surprising that non-bHLH TFs have also been implemented in forward programming of PSCs. Yamamizu et al. (2013) for instance, identified that doxycycline-mediated induction of Nr2f1 or Smad7 can instruct neuronal differentiation of mouse ESCs. Liu et al. (2018) further performed a comprehensive CRISPR activation screen in mouse ESCs and revealed that besides the bHLH TFs *Ngn1* and *Tcf15*, also non-bHLH TFs such as *Brn2*, *Foxo1*, *Ezh2* and *Zeb1* have neurogenic potential. Zeb1 and Zeb2, for instance, are homologous TFs and downstream effectors of the lncRNA-1604, which regulates neural differentiation by competitive binding with miRNA-200c in mouse ESCs (Weng et al., 2018), and *ZEB1* is also induced after combined overexpression of NGN1 and NGN2 in human iPSCs (Busskamp et al., 2014). Yet, at least for Zeb1 it was shown by other groups that its neuron-promoting effect is comparably weak. When overexpressed in human ESCs, ZEB1 does not immediately decrease pluripotency markers *OCT4, NANOG*, and *SOX2*, and most cells are negative for the early neuronal marker TUBB3 up until day 25 of differentiation (Jiang et al., 2018). Since it was demonstrated that Zeb2 overexpression decreases *Ngn2* expression in embryonic midbrain cells and a dopaminergic cell line (Yang et al., 2018), a negative correlation between these two TFs might account for the observed weak pro-neurogenic effect. Conversely, forward programming by *Ezh2* induction results in the formation of electrophysiological active and synapse-forming neurons, which is comparable to the effect of Ngn1-mediated forward programming. The pro-neurogenic action of Ezh2 might most likely be due to its inhibitory effect on endodermal and mesodermal lineage-associated genes (Liu et al., 2018), which is in line with the fact that the methyltransferase Ezh2 is a core component of the PRC2 complex and involved in transcriptional repression.

In sum, these studies hint at some common mechanisms underlying TF-driven specification of NPCs and PSCs into neurons, which include (i) exit of the original cell fate, (ii) repression of alternative lineage decisions, and (iii) activation of a pro-neuronal transcriptional program.

### Forward Programming Into Clinically Relevant Neuronal Subtypes

When thinking of forward programming as a tool to produce neural cell types for brain repair, it is particularly relevant to thoroughly characterize the exact phenotype of the obtained cells. Already Serre et al. (2012) noted that the four different bHLH TFs NGN1, NGN2, NGN3, and ASCL1 had slightly varying effects on neuronal subtype specification from human primary cortical NPCs, although cultures generally consisted of a mixed population of GABAergic, cholinergic, serotoninergic, adrenergic, and MNs (Serre et al., 2012). This observation is in line with other reports demonstrating divergent effects for different bHLH TFs on neuronal subtype derivation: Overexpression of ASCL1 induces a GABAergic bias in neuronal cultures differentiating from neurospheres isolated from both human fetal cortex and mesencephalon (Kim et al., 2009), whereas NGN2 overexpression in human iPSC-derived NPCs (Ho et al., 2016) and PSCs (Zhang et al., 2013; Nehme et al., 2018; Meijer et al., 2019; Rhee et al., 2019; Nickolls et al., 2020) leads to the derivation of mostly glutamatergic neurons.

#### Cortical Glutamatergic and Forebrain GABAergic Neurons

The results of multiple independent studies indicate that glutamatergic neurons derived by overexpression of NGN2 adopt a telencephalic fate characterized by the expression of cortical layer II/III markers such as FOXG1, BRN2, SATB2, and *CUX1* (Zhang et al., 2013; Frega et al., 2017; Wang et al., 2017; Nehme et al., 2018; Meijer et al., 2019). However, there are reports indicating that forward programming with a combination of NGN1 and NGN2 results in neurons co-expressing vGLUT1 and ChAT (Busskamp et al., 2014). Similarly, mRNA-driven combinatorial overexpression of NGN1, NGN2, NGN3, NEUROD1, and NEUROD2 in human PSCs gives rise to a population of remarkably pure cholinergic MNs (Goparaju et al., 2017), indicating that NGN2 can, in particular in combination with additional TFs and morphogens, instruct other fates than glutamatergic neurons (see also section ‘Motor and Sensory Neurons’).

Ascl1 – which is expressed in more ventral regions of the telencephalon *in vivo* (Casarosa et al., 1999; Fode et al., 2000) – is alone insufficient to consistently give rise to homogenous cultures of only one specific neuronal subtype and instead results in mixed cultures of MNs, dopaminergic and GABAergic neurons (Yamamizu et al., 2013). However, in a landmark study, Yang et al. (2017) demonstrated that overexpression of ASCL1 in combination with DLX2, a downstream effector of ASCL1, is able to direct human PSCs into remarkably pure cultures of telencephalic forebrain GABAergic neurons. Sun et al. (2016) used a combination of ASCL1 and DLX2 with LHX6 and a synthetic cluster of miRNA-9/9^∗^ and miRNA-124 (miR9/9^∗^-124), and found that the induced GABAergic neurons express markers reminiscent of derivatives of the medial ganglionic eminence, but not alternative birthplaces such as the lateral ganglionic eminence, caudal ganglionic eminence or the preoptic area. As somatostatin- and parvalbumin-positive inhibitory interneurons appear to play a particular role in several neurological and neuropsychiatric diseases, the generation of these subtypes would be highly desirable. Colasante et al. (2015) reported that combinatorial overexpression of the five TFs FOXG1, SOX2, ASCL1, DLX5, and LHX6 in human iPSCs gives rise to highly enriched cultures of parvalbumin-expressing inhibitory neurons. Yuan et al. (2018) explored overexpression of LHX6 alone and found that 80% of the human PSC-derived neurons were GABAergic, with a fraction of 21% and 29% of the TUBB3-positive neurons co-expressing parvalbumin and somatostatin, respectively. As with forward programmed excitatory neurons, functional maturation of induced GABAergic neurons can be promoted by co-culture with primary rodent glia (Sun et al., 2016). Interestingly, this process can also be promoted by co-culture with NGN2-forward programmed excitatory neurons (Yang et al., 2017) – an observation which could suggest that utmost functionality can only be achieved in the context of a heterogenous synaptic network as encountered *in vivo*.

#### Midbrain Dopaminergic Neurons

Representing a prime target of PD, midbrain dopamine neurons are a particularly attractive donor cell population for neural repair. However, the efficacy of neuroregeneration seems to heavily depend on the fidelity of neuronal subtype specification. This was recently exemplified by Kirkeby et al. (2017), who revealed that the purity of dopaminergic cell preparations (i.e., the ratio of caudal ventral mesencephalic dopaminergic neurons versus neurons of the diencephalic subthalamic nucleus) is predictive for successful dopaminergic specification and symptom amelioration after transplantation into a mouse model of PD. Although potent extrinsic factor-guided protocols for the derivation of dopaminergic neurons from PSCs exist (Kriks et al., 2011; Kirkeby et al., 2012), there is a necessity to further fine-tune cell fate subspecification. This was nicely illustrated by La Manno et al. (2016), who profiled the developing mouse and human midbrain using single cell RNA sequencing, and delineated multiple molecularly diverse NPC populations and several distinct classes of mature dopaminergic neurons in the midbrain of both species. Notably, the authors compared transcriptomic signatures of human PSC-derived dopaminergic neurons with their *in vivo* counterparts and found that although the morphogen-driven dopaminergic differentiation recapitulated key developmental stages of embryonic dopaminergic lineage specification, the gene expression profile of *in vitro* generated populations still differed from that of native midbrain dopamine neurons. The question remains whether forward programming can further improve the authenticity of specialized dopaminergic neuron subpopulations.

Already beginning in the end of last century, several labs reported that overexpression of the mesencephalic TF Nurr1 in primary adult rat hippocampal NPCs (Sakurada et al., 1999) and primary embryonic rat cortical NPCs (Kim et al., 2003; Park et al., 2006; Bae et al., 2009) promotes the generation of a midbrain dopamine neuron-like phenotype. A similar effect was communicated for primary mouse NPCs isolated from the ganglionic eminence and midbrain as well as mouse ESC-derived NPCs, whereas NPCs from embryonic cortex and spinal cord as well as adult NPCs from the subventricular zone seemed resistant to the pro-dopaminergic patterning effect of Nurr1 (Soldati et al., 2012). Other midbrain-specific TFs that were tested for their potency to instruct dopaminergic fates include (i) Foxa2, which, when overexpressed in mouse primary midbrain-derived and ESC-derived NPCs, was found to boost the derivation of TH-positive neurons (Kittappa et al., 2007), (ii) Lmx1a, which efficiently specifies murine Shh- and Fgf8-treated (Andersson et al., 2006; Panman et al., 2011) as well as human ESC-derived NPCs toward a dopaminergic fate (Friling et al., 2009), and in combination with Foxa2 and Barhl1 drives dopaminergic differentiation from Fgf8- and CHIR99021-exposed murine ESC-derived NPCs (Kee et al., 2017), (iii) En1 and (iv) Otx2, which were reported to drive dopaminergic differentiation of murine NPCs alone, each in combination with Lmx1a, or as a 3 TF cocktail (Panman et al., 2011), (v) Dmrt5, which does not increase overall neuronal yield after being overexpressed in mouse ESC-derived dopaminergic NPCs but specifically induces an increase of certain midbrain dopaminergic markers on RNA level (Gennet et al., 2011), and (vi) PBX1, which appears to cooperate with NURR1 promoting dopaminergic specification from human PSC-derived NPCs (Villaescusa et al., 2016). Another good example is the recently published study by Azimi et al. (2018), who used magnetically guided mRNA spot delivery to screen single TFs and TF combinations for their capacity to commit human ESC-derived NPCs toward a dopaminergic fate, and revealed that transfection of FOXA2, LMX1A, and PITX3 mRNA results in an increased yield of TH-positive neurons. Combinatorial delivery of these 3 TFs at their respective most effective stage results in almost 68% of TH- and MAP2-double-positive cells (Azimi et al., 2018).

Nurr1 was also the first TF explored in the context of forward programming PSCs toward a dopaminergic fate, and its overexpression in mouse ESCs causes a substantial increase in the number of TH-expressing neurons (Chung et al., 2002; Kim et al., 2002). Exposure to the morphogens Shh and Fgf8 further increases the yield of TH-expressing neurons after Nurr1 overexpression with enrichment in the order of 60% to 80% (Chung et al., 2002; Kim et al., 2002), and enhances the release of dopamine after induced depolarization (Kim et al., 2002). The combination of Nurr1 with other TFs (such as Gpx1), morphogens (as for instance RA) and chemicals (e.g., β-boswellic acid) was reported to boost the yield of dopaminergic neurons, too (Abasi et al., 2012). Especially the combined expression of Nurr1 and Pitx3 was shown to be beneficial for the derivation of dopaminergic neurons from mouse PSCs (Martinat et al., 2006; Salemi et al., 2016). Whereas these TFs alone are sufficient to induce markers expressed early in dopaminergic development such as TH and *Aldh2*, it is only upon co-expression that they synergistically induce more advanced markers such as *Dat* and *Tyrp1* (Martinat et al., 2006). In this co-expression paradigm, too, addition of Shh and Fgf8 increased the induced secretion of dopamine from these cells (Salemi et al., 2016). Notably, and in contrast to overexpression of Nurr1 alone (Kim et al., 2002), co-expression of Nurr1 and Pitx3 (along with exposure to Shh and Fgf8) prevents the derivation of ‘contaminating’ cell fates such as serotonergic or GABAergic neurons (Martinat et al., 2006).

Sánchez-Danés et al. (2012) analyzed the effect of LMX1A overexpression in human iPSCs and observed a quick down-regulation of *NANOG* with a simultaneous upregulation of *NURR1*, *EN1*, and *TH*, which are all characteristic markers of midbrain dopamine neurons. At day 34 of differentiation, the derived neurons express DAT and TH, show synaptophysin-positive puncta on TH-positive neurites and inducible dopamine release. Although LMX1A overexpression alone enhances the dopaminergic specification of differentiating neurons, it does not result in an increased neuronal yield (Sánchez-Danés et al., 2012).

The group of Vania Broccoli explored forced expression of LMX1A together with NURR1 and ASCL1 in order to boost neuronal induction *per se* as well as dopaminergic induction in particular. In their study, lentiviral overexpression of this TF cocktail in human iPSCs gave rise to homogenous neuronal cultures expressing a wide range of proteins associated to the dopaminergic lineage such as TH, DAT, ALDH1A1, and GIRK2. Resulting neurons exhibited neuron-like electrophysiological properties and were able to spontaneously release dopamine after 3 weeks of differentiation. However, although the number of TH- and TUBB3-co-expressing cells doubled when applying the TF cocktail (Theka et al., 2013), the overall yield of TH-expressing neurons remained lower as compared to the yield after overexpression of LMX1A alone (Sánchez-Danés et al., 2012). This difference might be attributable to the lack of additional patterning molecules such as SHH and FGF8 in the medium used by Vania Broccoli’s group, and/or shorter differentiation times till analysis, and thus warrants further studies on forward programming approaches combining neurogenic and regionalizing TFs.

Finally, it is worth mentioning that the bHLH TF ATOH1 is itself able to induce dopaminergic neurons from human PSCs, especially when combined with exposure to SHH and FGF8b. In combination with these morphogens, ATOH1 overexpression yields up to 82% TH-expressing neurons. In addition to *TH*, *FOXA2*, *NURR1*, *LMX1A*, *DAT*, *VMAT2*, and *OTX2* are significantly upregulated during ATOH1-driven differentiation. Characterization of the growth factor-treated, ATOH1-overexpressing neuronal cultures on a functional level demonstrated that these neurons possess electrophysiological properties similar to primary rat midbrain dopaminergic neurons and exhibit dopamine release after electrical stimulation at day 36 of differentiation, suggesting actual functionality (Sagal et al., 2014). Very recently, this approach was further improved by establishing a protocol based on combined ATOH1 and NGN2 overexpression in human iPSCs via repetitive mRNA transfections (Xue et al., 2019).

#### Medium Spiny Neurons

Another neuronal subtype of particular biomedical interest are MSNs, a GABAergic population abundantly found in the striatum and most prominently affected by HD. In human ESC-derived NPCs treated with SHH and the WNT inhibitor DKK1, overexpression of GSX2 and EBF1, two TFs essential for the development of striatal interneurons, actively suppresses the expression of the medial ganglionic eminence progenitor markers PAX6 and NKX2.1 and drives cell cycle exit. After 60 days of long-term differentiation, overexpression of both TFs finally results in MSN progenitor cells expressing ISL1- and CTIP2; by day 80, 38.8% of all cells co-express the MSN markers DARPP32 and CTIP2 (Faedo et al., 2017). However, whether or not this TF combination would be capable of directly specifying undifferentiated PSCs to MSNs merits further investigation.

#### Motor and Sensory Neurons

Motor neuron development and specification *in vivo* is relatively well studied (see reviews by Jessell, 2000 and Briscoe and Ericson, 2001), and this knowledge was efficiently exploited for studies focusing on the *in vitro* generation of enriched MN populations, which are affected by degenerative diseases such as amyotrophic lateral sclerosis. Hester et al. (2011) for instance, successfully combined NGN2-driven differentiation of SHH- and RA-treated human PSC-derived NPCs with overexpression of the MN lineage-specific markers ISL1 and LHX3 yielding 60% cells co-expressing the MN markers ISL1 and ChAT at day 13 of differentiation. In 2013, the group of Hynek Wichterle demonstrated that overexpression of this TF combination can also successfully specify the differentiation of mouse ESCs into spinal MNs directly, whereas cranial MNs can be obtained by replacing Lhx3 by Phox2a in this TF cocktail. A systematic comparison of these two different TF combinations by gene array analysis revealed a sharp decrease of pluripotency-associated genes *Oct4* and *Nanog* and an upregulation of pan-MN markers such as *Isl1*, *Ebf1* and *Ebf3* as well as the VAChT in both paradigms, whereas upregulation of *Tbx20*, *Phox2a*, *Phox2b*, *Rg4*, and *Gal* was only detected in neurons subjected to forward programming with Phox2a. The results of this study further indicate that these divergent outcomes are obtained because Isl1 is recruited to different genomic sites when co-expressed with Ngn2 in combination with either Lhx3 or Phox2a. Yet, both MN subpopulations become electrophysiologically functional and capable of forming cholinergic synapses after maturation on cortical mouse astrocytes (Mazzoni et al., 2013). A few years later, Goto et al. (2017) verified that Sendai virus-mediated overexpression of the TF cocktail NGN2, ISL1 and LHX3 in human PSCs, too, promotes the expression of MN markers. More specifically, only the full TF cocktail and the 2-factor combination of NGN2 and LHX3 but neither NGN2 in conjunction with ISL1 nor any of the single TFs resulted in MN derivation. Notably, after 3 weeks of differentiation NGN2/ISL1/LHX3-overexpressing neurons were electrophysiologically active and formed neuromuscular junctions with cultured myocytes (Goto et al., 2017). De Santis et al. (2018) expressed both TF combinations identified by Hynek Wichterle’s group (NGN2/ISL1/LHX3 and NGN2/ISL1/PHOX2A) in human iPSCs via Piggy-bac transposable vectors. Concordant with the previous results, iPSCs downregulated the pluripotency marker *NANOG* and upregulated pan-MN genes such as *TUBB3*, *ISL1*, and *ChAT* within the first 3 days of differentiation. By day 5, *HB9* expression was increased when LHX3 was co-expressed, whereas *PHOX2B*, *TBX20*, and *RG4* were detected upon PHOX2A overexpression. Finally, the authors of this study functionally characterized the cranial MNs obtained after 12 to 13 days of NGN2/ISL1/PHOX2A overexpression and observed that these cells were capable of firing action potentials upon current stimulation, and almost half of all analyzed cells even displayed spontaneous glutamatergic postsynaptic currents (De Santis et al., 2018).

Whilst these studies utilized joined overexpression of the neurogenic TF Ngn2 with MN lineage-associated TFs, Goparaju et al. (2017) investigated whether overexpression of generic neurogenic TFs can induce specified neuronal subtypes when combined with fate-modulating extrinsic factors. Indeed, they found that overexpression of NGN1, NGN2, NGN3, NEUROD1, and NEUROD2 in human PSCs combined with RA, forskolin and dual SMAD inhibition via SB431542 and dorsomorphin yields highly pure neuronal cultures expressing the MN markers HB9, ISL1, and ChAT (Goparaju et al., 2017). Interestingly, the combination of NGN2 overexpression with forskolin and dorsomorphin treatment has been described to even convert human fibroblasts into cholinergic MNs (Liu et al., 2013).

On the other hand, Panman et al. (2011) revealed that overexpression of the MN-associated TFs Phox2b and Olig2 in mouse ESC-derived, posterior-ventral NPCs suffices to specify visceral and somatic MNs, respectively. Further dissecting the role of Phox2 TFs in segregating MNs from other populations of hindbrain neurons, Mong et al. (2013) overexpressed either Phox2a or Phox2b in Nestin-expressing ESC-derived NPCs. Although the expression of both TFs largely overlaps *in vivo*, the sequence of their expression is known to be important for the specification of different neuronal subtypes: Phox2a precedes Phox2b induction during the development of noradrenergic (Pattyn et al., 2000) and midbrain MNs (Pattyn et al., 1997), whereas in hindbrain visceral MNs, Phox2b is induced before Phox2a (see review by Brunet and Pattyn, 2002). Knock-out studies further showed that although Phox2b expression can compensate for effects caused by Phox2a-knock-out in noradrenergic neurons of the locus coeruleus, it does not suffice to rescue the loss of MNs in the midbrain (Coppola et al., 2005). *Vice versa*, Phox2a cannot completely compensate for Phox2b loss during the development of noradrenergic neurons and visceral MNs (Coppola et al., 2005). Concordant with these findings, overexpression of both Phox2 TFs in mouse and human ESC-derived NPCs upregulated the expression of visceral MN markers as for instance *Isl1*, *Nkx6.2*, *Tbx2*, and *Tbx20* when combined with the morphogens Fgf8 and Shh. Conversely, combining Phox2b but not Phox2a overexpression with Bmp7 and Fgf8 treatment increased the expression of genes characteristic for noradrenergic neurons such as *Tfap2a*, *Dbh*, *Tlx3*, and *Net* (Mong et al., 2013).

Very recently, the group of Carsten Bönnemann published a protocol to derive sensory neurons from human iPSCs via forward programming. The authors demonstrated that even in the absence of neuronal lineage-promoting medium conditions, doxycycline-induced expression of NGN2 and BRN3A from the human genomic safe harbor locus *CLYBL* specifies human iPSCs toward a presumable human-specific neuronal subtype of glutamatergic sensory neurons responsive to cold as well as mechanical stimuli. This neuronal phenotype was also acquired when expression of this TF combination was induced in iPSC-derived neural crest progenitors for 14 days. Notably, when iPSC-derived neural crest progenitors were exposed to doxycycline for as short as 24 h, these cells adopted an exclusively PIEZO2-positive but TRPM8-negative touch-sensitive phenotype (Nickolls et al., 2020). This finding is in line with the observation that even a 24-h pulse of NGN2-only overexpression (in combination with a GDNF-based differentiation paradigm) is sufficient to direct human ESC-derived neural crest cells into highly enriched cultures of mechanoreceptive neurons (Schrenk-Siemens et al., 2015).

In sum, whilst these studies impressively illustrate the potential of the forward programming technique to derive distinct neuronal subtypes, they also demonstrate the sensitivity of the approach to subtle alterations in TF combinations and co-administered growth and patterning factors.

### Glial Cells

Astrocytes are crucial for neuronal development, synaptogenesis and synaptic function, brain tissue homeostasis including energy and substrate distribution, and they provide the structural scaffold of the brain parenchyma. Oligodendrocytes are not only crucial for myelination but also axonal maintenance and even immunomodulation (reviewed by Kuhn et al., 2019). Given the plethora of glial functions that are essential for proper brain physiology, the role of these cells in the pathogenesis of neurodegenerative diseases becomes increasingly acknowledged, which contributes to the great interest in producing glial cells for basic and translational research in a fast and efficient manner by TF overexpression (Table 2).

**TABLE 2:** Transcription factors used for promoting glial differentiation of neural precursor cells and pluripotent stem cells *in vitro*.

Derived cell type	Starting cell type	Species	Transcription factor used for forward programming	References
Astrocytes	NPCs	Mouse	Emx2	Falcone et al. (2015)
	NPCs	Mouse	Rnf20	Liang et al. (2018)
	iPSC-derived NPCs	Human	NFIA	^(17)^ Tchieu et al. (2019)
	PSCs	Human	NFIB or NFIB+SOX9	^(18)^ Canals et al. (2018)
	PSCs	Human	NFIA or NFIA+SOX9	^(16)^ Li X. et al. (2018)
Oligodendrocytes	NPCs	Mouse	Olig1	Balasubramaniyan et al. (2004)
	NPCs	Mouse	Olig1 or Olig2 or Nkx2.2	^(19)^ Copray et al. (2006)
	NPCs	Mouse	Ascl1 or Olig2 or Nkx2.2 or Ascl1+Olig2 or Ascl1+Nkx2.2 or Olig2+Nkx2.2	Sugimori et al. (2008)
	NPCs	Mouse	Nkx2.2AS	Tochitani and Hayashizaki (2008)
	NPCs	Mouse	Olig1 or Olig2	Maire et al. (2010)
	NPCs	Mouse	Ascl1 or Olig2 or Sox10	Braun et al. (2015)
	NPCs	Mouse	Sox10	^(24)^ Matjusaitis et al. (2019)
	NPCs	Mouse	Lnc-158	Li Y. et al. (2018)
	NPCs	Human	OLIG1 or OLIG2 or OLIG1+OLIG2	^(21)^ Hwang et al. (2009)
	NPCs	Human	OLIG2	^(20)^ Maire et al. (2009)
	NPCs	Human	ASCL1 or NKX2.2 or OLIG2 or PRRX1 or SOX10	^(22)^ Wang et al. (2014)
	NPCs	Human	OLIG1 or OLIG2 or OLIG1+OLIG2	Li et al. (2017)
	iPSC-derived NPCs	Human	SOX10 or NKX6.2+OLIG2+SOX10	^(23)^ Ehrlich et al. (2017)
	PSC-derived NPCs	Human	SOX10	^(25)^ García-León et al. (2018)
	iPSCs	Human	OLIG2+SOX10	Li P. et al. (2016)
	PSCs	Human	SOX10 or OLIG2+SOX10	Pawlowski et al. (2017)

*Numbers in superscript relate to citations in Figure 2.*

#### Forward Programming to Astrocytes

As with neurons, astrocytes – and even further specified astrocyte subtypes – can be differentiated from PSCs by multi-step, growth factor-based protocols, which stimulate signaling pathways involved in astrogenesis after initial induction of a neuroectodermal fate (compare, e.g., the elegant protocol by Krencik and Zhang, 2011). Yet, growth factor-based protocols are usually complex and time-consuming, especially if they are aiming at creating non-reactive cells resembling quiescent astrocytes *in vivo*. Hence, there is a need for the derivation of functional astrocytes via forward programming. In primary mouse cortical NPCs, astrocytic commitment can be facilitated via induction of Stat3 by overexpression of Rnf20 (Liang et al., 2018). Overexpression of the TF Emx2 in mouse cortical NPCs regulates Egf and Fgf signaling, which are crucial for maintaining the pool of proliferating astrocyte progenitors (Falcone et al., 2015).

In a landmark study, Canals et al. (2018) lentivirally overexpressed the NFI TF family member NFIB alone or in combination with SOX9 in human PSCs. In this paradigm, PSCs differentiate into mature, post-mitotic astrocytes expressing markers such as GFAP, S100β, VIM, ALDH1L1, and GLAST within 21 days of differentiation. At this differentiation stage, astrocytes further contain glycogen-positive granules comparable to primary astrocytes. Functional assessments of these forward programmed astrocytes between days 14 and 21 of differentiation revealed that the derived cells exhibit typical characteristics of human adult astrocytes such as generation and propagation of spontaneous calcium waves, glutamate uptake, responsiveness to ATP and inflammatory stimuli such as IL-1β, the ability to form functional gap junctions with other astrocytes and the potency to promote synaptogenesis in a co-culture system with iPSC-derived forward programmed neurons (Canals et al., 2018).

Independent of and almost at the same time as the report by Canals et al. (2018), the lab of Su-Chun Zhang published a protocol to derive functional astrocytes from human PSCs by doxycycline-inducible expression of NFIA, another NFI TF family member, via CRISPR/Cas9-mediated targeting of the human *AAVS1* genomic safe harbor locus. By overexpressing SOX9 in addition to NFIA, and combining this forward programming protocol with a conventional morphogen-driven astrocyte differentiation paradigm, the efficiency of astrocyte generation was significantly increased so that finally around 70% of all cells co-expressed the astrocyte markers GFAP and S100β at day 52 of differentiation. Similar to the astrocytes derived by Canals et al. (2018), their cells could propagate calcium waves, take up free glutamate from the culture medium, and facilitate neurite outgrowth when co-cultured with human iPSC-derived neurons. Interestingly, the authors further reported that transgene induction during the first 10 days of differentiation was dispensable for successful astrocyte induction. Importantly, when they used this transgene induction-free window for morphogen-based patterning, they could generate diverse astrocytic subtypes (i.e., dorsal and ventral forebrain astrocytes as well as spinal astrocytes) within the same time frame (Li X. et al., 2018).

Despite the many similarities between the protocols published by Canals et al. (2018) and Li X. et al. (2018), it is noteworthy that the former protocol leads to the derivation of functional astrocytes much faster than the latter one (2–3 versus >7 weeks). This might have several causes, including the choice of the TFs itself (NFIB versus NFIA), the methods used for TF delivery that could influence total gene dosage (lentiviral expression versus expression from the endogenous *AAVS1* locus) and the efficiency of the concomitant growth factor regimen (e.g., sequential versus combined exposure to FGF and EGF). Thus, the results of these two studies stress the context-dependency of TF-based forward programming. This is further nicely exemplified by the fact that NFIA was recently demonstrated to act as a gliogenic switch in iPSC-derived NPCs, too, facilitating the fast generation of astrocytes in combination with glia-promoting, LIF-containing medium. Continued overexpression of this TF, however, inhibited astrogenesis from NPCs, probably by inducing premature G1 cell cycle arrest (Tchieu et al., 2019). Surprisingly, overexpression of the long non-coding RNA lnc-158, which is an endogenous antisense RNA of *NFIB* and positively regulates NFIB levels, has been reported to promote the differentiation of primary mouse NPCs into oligodendrocytes instead of astrocytes (Li Y. et al., 2018).

#### Promoting Oligodendrogenesis by Transcription Factor Overexpression

Wang et al. (2014) screened 5 TFs (NKX2.2, OLIG2, PRRX1, ASCL1, and SOX10) known to be associated with oligodendrocyte lineage commitment and analyzed their potency to induce OPC markers in primary human NPCs. Whilst all examined TFs repressed astrocytic genes, NKX2.2 and especially ASCL1 induced the expression of neuronal genes in addition to the upregulation of OPC markers. Gene set enrichment analyses of RNA sequencing data further revealed that only SOX10 overexpression induced genes expressed in both primary mouse and human OPCs, whereas ASCL1-induced OPCs expressed markers resembling mouse but not human OPC fate. The authors further demonstrated the superiority of SOX10-induced OPCs by the fact that only this population could be cultured *in vitro* for several passages whilst maintaining its oligodendrocyte differentiation potential (Wang et al., 2014). In line with the results of Wang et al. (2014) are various other reports from different groups demonstrating the oligodendrocyte-promoting effects of Ascl1 in combination with Olig2 or Nkx2.2 (Sugimori et al., 2008), as well as Ascl1 (Braun et al., 2015), Olig1/2 (Balasubramaniyan et al., 2004; Copray et al., 2006; Sugimori et al., 2008; Hwang et al., 2009; Maire et al., 2009, 2010; Braun et al., 2015; Li et al., 2017), Nkx2.2 (Copray et al., 2006; Sugimori et al., 2008; Tochitani and Hayashizaki, 2008) and Sox10 (Braun et al., 2015; Ehrlich et al., 2017; García-León et al., 2018; Matjusaitis et al., 2019) alone. Interestingly, overexpression of Ascl1 *in vivo* has also been shown to coax endogenous hippocampal NPCs into an oligodendroglial, myelination-competent phenotype (Jessberger et al., 2008; Braun et al., 2015).

Ehrlich et al. (2017) demonstrated that overexpression of SOX10 alone can induce oligodendrocyte differentiation of cultured human iPSC-derived NPCs. Yet, oligodendrocyte derivation is more efficient when SOX10 is overexpressed in combination with OLIG2 and NKX6.2. With this improved protocol, around 60% to 80% of all cells stain positive for GALC and O4 at day 28 of differentiation and O4-enriched oligodendrocytes exhibit the capability to myelinate iPSC-derived neurons after 3 weeks of *in vitro* co-cultivation (Ehrlich et al., 2017). One year after the report of Ehrlich et al. (2017), also the group of Catherine Verfaillie published a protocol to derive oligodendrocytes from human PSC-derived NPCs by lentiviral SOX10 overexpression: García-León et al. (2018) performed RNA sequencing analysis of purified O4-positive cells at day 22 of differentiation, demonstrating that their protocol gives rise to oligodendrocytes that highly resemble intermediate to mature primary human brain-derived oligodendrocytes. Moreover, purified O4-positive oligodendrocytes were able to myelinate human iPSC-derived neurons after 20 days of co-culture. Finally, García-León et al. (2018) created a stable human ESC-line with doxycycline-inducible expression of SOX10 from the endogenous *AAVS1* locus and demonstrated that this approach successfully generates mature oligodendrocytes when transgene expression is induced at the NPC stage. Noteworthy, however, doxycycline-induced expression of SOX10 at the ESC stage was insufficient to give rise to MBP-expressing oligodendrocytes (García-León et al., 2018).

Whereas these studies have identified multiple routes to promote oligodendrocyte differentiation from an NPC stage, direct TF-driven specification of PSCs toward the oligodendrocyte lineage has remained more challenging to achieve. Li P. et al. (2016) reported that combining a multi-step growth factor-based differentiation protocol with SOX10 and OLIG2 overexpression in human iPSCs results in cultures consisting of around 40% O4-positive oligodendrocytes after 4 weeks of differentiation. However, when co-culturing SOX10/OLIG2-induced OPCs with embryonic primary rat cortical neurons, only around 5% of all cells stained positive for O4 at day 14 of co-culture and just 0.5% of all rat axons co-labeled with processes extending from human forward programmed oligodendrocytes (Li P. et al., 2016). One year later, the group of Mark Kotter published a highly controlled SOX10 and OLIG2-driven forward programming protocol for the derivation of oligodendrocytes from human PSCs, which is based on inducible transgene overexpression by dual genomic safe harbor targeting (i.e., targeting the doxycycline-responsive transcriptional activator to the *ROSA26* and the tetracycline-responsive element-regulated transgenes of interest to the *AAVS1* locus). Interestingly, using this system, the authors could generate proliferative OPCs, which terminally differentiated into almost pure cultures of oligodendroglial cells expressing characteristic markers such as CNP and PLP upon mitogen withdrawal (Pawlowski et al., 2017).

It will be interesting to investigate whether TFs and TF combinations explored in the context of fibroblast-to-glia transdifferentiation can be exploited for forward programming of PSCs. For instance, direct cell fate conversion of rodent fibroblasts into OPCs was achieved by combined overexpression of the TFs Sox10 and Olig2 with either Nkx6.2 (Najm et al., 2013; Matjusaitis et al., 2019) or Zfp536 (Yang et al., 2013). Similarly, myelination-competent Schwann cells can be derived from mouse and human fibroblasts via the combined overexpression of Sox10 and Krox20 (Mazzara et al., 2017; Sowa et al., 2017) and have been shown to accelerate nerve regeneration and motor recovery after transplantation into mice with sciatic nerve transection (Sowa et al., 2017).

Taken together, the results of these studies underpin the potency of some TFs and/or TF combinations for oligodendrocyte specification. Yet, the efficient direct specification of oligodendrocytes form a PSC state deserves further attention. In addition to TFs, miRNAs could be supportive in this process. The miRNAs miR-219 and miR-338 were shown to promote oligodendrocyte maturation by targeting inhibitors of oligodendrogenesis such as Sox6 and Hes5, as well as promoters of neurogenesis as for instance Neurod1, Isl1, Otx2, and Zfp238/RP58 (Zhao et al., 2010).

## *In vivo* Application of Forward Programmed Cells: Insights From Transplantation Studies

As a proof-of-concept for the general applicability of forward programmed cells for neuroregenerative approaches, these cells can be transplanted into healthy animals and monitored for graft survival, maturation and integration (Figure 2). Along this line, Zhang et al. (2013) demonstrated that 6 weeks after transplanting human immature neurons (7 days after infecting ESCs with a lentivirus encoding for NGN2) into the mouse striatum, the grafted cells adopted a neuronal phenotype exhibiting dendritic arborizations, axonal outgrowth and electrophysiological functionality, and received inhibitory synaptic input from host striatal interneurons (Zhang et al., 2013). Yuan et al. (2018) transplanted human PSC-derived NPCs, forward programmed by LHX6 overexpression, into the ventral mouse forebrain. Surprisingly, the authors observed that the number of GABAergic interneurons overall was not significantly increased in the doxycycline-induced, forward programmed grafts as compared to uninduced control transplants, and LHX6-overexpressing as well as uninduced control grafts differentiated into all four interneuron subtypes. Still, the forward programmed neurons exhibited spontaneous electrophysiological activity and postsynaptic currents reflecting mostly inhibitory GABAergic input (Yuan et al., 2018), demonstrating that forward programmed neurons can – in principal – exhibit proper functionality upon grafting. This notion is substantiated by reports of several other groups: For instance, 14-day-old neurons, derived from human PSCs by ASCL1- and DLX2-overexpression, survive transplantation into the subventricular zone and cerebral cortex of neonatal mice and mature into GABAergic neurons within 3 months post transplantation (Yang et al., 2017), and neurons derived from human PSCs by combined ASCL1, DLX2 and LHX6 overexpression mature into GABAergic neurons *in vivo*, too (Sun et al., 2016). Notably, 2 months after grafting, these GABAergic neurons had functionally integrated into cortical layers V and VI, exhibiting repetitive action potential firing and receiving synaptic input from host neurons (Sun et al., 2016). In an elegant study by the group of Hynek Wichterle, MNs were programmed by overexpressing Ngn2 and Isl1 in combination with either Lhx3 or Phox2a in mouse ESCs, and the resulting cells were grafted into the cervical and brachial tube of chicken embryos 2 days after transgene induction. Already 2 days after transplantation, the grafted cells had spatially segregated and exhibited axonal projections concordant with their MN subclass identity: Like spinal MNs, Ngn2/Isl1/Lhx3-overexpressing cells accumulated in axial and limb nerve branches and exhibited substantial axonal outgrowth from the ventral root of the spinal cord, whereas Ngn2/Isl1/Phox2a-derived neurons accumulated in the lateral spinal cord and projected axons toward the spinal accessory nerve resembling cranial MNs (Mazzoni et al., 2013).

Whilst transplantation into unlesioned healthy recipients can be a highly useful tool to assess the *in vivo* differentiation and function of forward programmed neurons, studies in the context of a disease model can provide information on their regenerative capacity. First milestones to use forward programmed neurons for experimental neuroregeneration were already achieved as early as 2002, when Kim et al. (2002) transplanted Nurr1-overexpressing mouse ESC-derived neurons in the striatum of 6-OHDA-lesioned rats, an animal model of PD. 4 to 8 weeks post transplantation, the majority of transplanted cells expressed the dopaminergic marker TH, and 5 out of 6 grafts exhibited spontaneous postsynaptic currents. Most importantly, the authors demonstrated that animals transplanted with Nurr1-overexpressing neurons showed improved behavioral recovery compared to animals receiving sham injections or grafts of wild-type cells (Kim et al., 2002). Martinat et al. (2006) reported a few years later that Nurr1/Pitx3-induced mouse and human ESC-derived NPCs grafted into the striatum of 6-OHDA-lesioned mice resulted in a significant reduction in apomorphine-induced rotation behavior compared to the transplantation of control vector-transduced cells. However, further immunohistochemical analyses of the grafts revealed that in their setting, neurons retained an immature morphology with only a minority of them expressing TH (Martinat et al., 2006). In accordance with this finding, Theka et al. (2013) more recently showed that 12 days after transplanting immature dopaminergic neurons (8 days post inducing ASCL1, NURR1 and LMX1A in human iPSCs) 4 out of 6 grafts survived, and only a fraction of the surviving cells displayed neuronal morphologies and expression of TH (Theka et al., 2013). Similar results were reported by Kim et al. (2003), who demonstrated that although transplantation of rat wild-type midbrain NPCs improves behavior of 6-OHDA-lesioned rats, transplantation of Nurr1-overexpressing midbrain or cortical NPCs does not, presumably because Nurr1-NPC grafts contained fewer TH-positive neurons which additionally exhibited immature morphologies (Kim et al., 2003). Whilst these findings were confirmed by Park et al. (2006), their study further revealed that 8 weeks after transplanting rat NPCs overexpressing a combination of Nurr1, Ascl1 and Shh or Nurr1, Bcl-XL and Shh, dopaminergic specification and dopamine levels are increased and motor deficits decreased compared to transplantation of NPCs overexpressing Nurr1 alone (Park et al., 2006). In a study by Friling et al. (2009) only 50% of all grafts survived after transplanting mouse Lmx1a-overexpressing ESC-derived NPCs into 6-OHDA-lesioned rats. In these grafts, the majority of the transplanted cells co-expressed the dopaminergic markers TH, Pitx3, En1/2, Lmx1a and Vmat, and even non-overlapping positivity for Girk2 and calbindin, indicating generation of both, substantia nigra A9 neurons and ventral tegmental area A10 dopaminergic neurons (Friling et al., 2009). Lastly, NPCs derived from ESCs via forward programming with Lmx1a differentiate into TH, DAT and GIRK2-expressing dopaminergic neurons *in vivo*, too (Sánchez-Danés et al., 2012).

In addition to PD, HD is intensely explored as a candidate disease for neural cell replacement. Since striatal MSNs are the main target of the disease, fast and efficient *in vitro* generation of MSNs is a key prerequisite for a cell therapeutic approach. Faedo et al. (2017) grafted human ESC-derived NPCs carrying inducible GSX2 and EBF1 transgenes into the quinolinic acid-lesioned striatum and observed that 2 months after transplantation, these NPCs had differentiated into GABAergic neurons expressing the striatal MSN markers CTIP2 and DARPP32 and extending projections toward the substantia nigra (Faedo et al., 2017). However, comparable to what has been observed after transplantation of forward programmed dopaminergic neurons (Theka et al., 2013), the number of CTIP2-positive human neurons was not different in GSX2/EBF1-overexpressing transplants versus uninduced control grafts (Faedo et al., 2017). It remains to be investigated whether or not MSNs directly forward programmed from the PSC stage would survive and integrate upon transplantation.

Forward programmed glial cells might be valuable for neuroregenerative interventions, too. For astrocytes, however, there are only few published reports about the general feasibility of grafting these cells. Yet, these reports demonstrated that forward programmed astrocytes maintain their cellular identity up to 3 months after grafting (Li Y. et al., 2018a; Tchieu et al., 2019) and exhibit astrocyte-specific traits *in vivo* such as their affinity to blood vessels and the formation of functional gap junctions with host astrocytes (Canals et al., 2018). For oligodendrocytes, Copray et al. (2006) reported that Olig2-expressing mouse NPCs grafted into the demyelinated mouse striatum differentiate into mature MBP-positive oligodendrocytes engaging in remyelination (Copray et al., 2006). In line with this, Hwang et al. (2009) demonstrated that OLIG2-expressing NPCs survive transplantation into contused spinal cord better than wild-type NPCs and exhibit increased proliferation and migration into white matter tissue, where they efficiently differentiate into MBP-positive oligodendrocytes promoting myelination. Potentially due to this pro-myelinating effect, transplantation of OLIG2-NPCs improves locomotion after contusive injury compared to sham injection or transplantation of wild-type NPCs (Hwang et al., 2009). Also in Shiverer/Rag mice, which are used as models for myelination disorders, NPCs overexpressing OLIG2 (Maire et al., 2009) or SOX10 (Wang et al., 2014; Matjusaitis et al., 2019) have been shown to differentiate into MBP-positive oligodendrocytes exhibiting ensheathment of host axons. Transplantation of more mature O4-positive oligodendrocytes, which were derived from human NPCs by SOX10 overexpression, promotes myelination in brain slices of Shiverer/Rag mice as well (García-León et al., 2018). In an elegant study, Ehrlich et al. (2017). demonstrated that magnetic activated cell sorting (MACS)-purified O4-positive oligodendrocytes (14 days after induction of the TFs SOX10, OLIG2, and NKX6.2 in human NPCs) not only promote the formation of normally compacted MBP-positive sheaths with nodal structures around host neurons 16 weeks post transplantation but are also capable of remyelination after neurotransplantation in Shiverer/Rag mice treated with the membrane-dissolving chemical lysophosphatidyl-choline, which induces completely demyelinated lesions in white matter tissue (Ehrlich et al., 2017). However, since all of these transplantation studies were conducted with OPCs or oligodendrocytes derived by TF overexpression in NPCs, there is no proof so far that oligodendrocytes forward programmed from PSCs can survive neurotransplantation. This certainly merits further investigations, since OPCs directly converted from somatic fibroblasts were demonstrated to be capable of differentiating into myelinating oligodendroglial cells after transplantation into the brain of Shiverer/Rag mice, too (Najm et al., 2013; Yang et al., 2013).

## Forward Programming of PSCs Versus Primary Cell Fate Conversion

Since forward programming of PSCs can be regarded as a fallout of the technological advances of somatic cell reprogramming into iPSC and direct interconversion of somatic cells within and across germ layers, it is interesting to reflect on the commonalities and differences of these *in vitro* approaches. From a mechanistic point of view, direct cell fate conversion can be segregated into two different phases, which has been nicely deciphered in several milestone publications in the context of transdifferentiating fibroblasts into neurons via Ascl1, Brn2, and Myt1l overexpression (Vierbuchen et al., 2010; Wapinski et al., 2013; Treutlein et al., 2016; Mall et al., 2017). Here, the fibroblast’s chromatin landscape has to be remodeled first in order to become permissive to TF binding at neuron-specific genes. This chromatin opening can be mediated by small molecules acting as epigenetic modifiers, or induced by cell type-specific pioneer TFs, which – by definition – are able to bind to and open up closed chromatin. Second, following the necessary epigenetic rearrangements, the transcriptional landscape has to be modulated in order to activate neuronal genes and inhibit the acquisition of alternative fates, including the repression of fibroblast-specific transcriptional signatures. Notably, many principles regulating the acquisition of a new cell fate during transdifferentiation seem to apply to forward programming, too. Aydin et al. (2019) recently analyzed how Ngn2 and Ascl1 specify mouse ESCs into neurons and demonstrated that both bHLH TFs act as neuronal pioneer TFs binding to genes which are in closed chromatin states in ESCs. By this, Ngn2 and Ascl1 induce and recruit secondary pro-neural TFs such as Brn2, thereby promoting the acquisition and stabilization of a neuronal fate. Interestingly, and in accordance with other publications, Aydin et al. (2019) further report that despite the high similarity of the mechanistic action of these two TFs, Ngn2 and Ascl1 induce quite distinct neuronal programs as the binding patterns of both TFs in ESCs are largely divergent.

Although the general principles underlying forward programming and direct cell fate conversion seem to be quite similar, there are some differences which need to be highlighted (Table 3). First, the epigenetic hurdles that have to be overcome for the proper activation of an alternative transcriptional program seem to be lower in PSCs than in terminally differentiated somatic cells (in particular in the case of a trans-germ layer conversion). Thus, although Ascl1, for instance, is sufficient to specify PSCs into neurons, it is comparably inefficient to convert fibroblasts into authentic iNs when overexpressed alone (Liu et al., 2018), and Ngns or Neurods, which are commonly used in forward programming paradigms, seem almost incapable of converting fibroblasts into iNs (Chanda et al., 2014; Liu et al., 2018). Second, from a time perspective, the derivation of neuronal cells from somatic cells is faster via the direct conversion route compared to forward programming via the iPSC stage: In this scenario, transdifferentiation is a one-step-procedure, whereas for forward programming, somatic cells have to be first reprogrammed into iPSCs before they can be differentiated into the desired somatic cell type by TF overexpression. However, due to the intermediate pluripotent stage, the forward programming route is scalable at the iPSC stage and can give rise to highly homogeneous cell batches. Notably, it is still not completely resolved whether forward programming of PSCs involves a stable NPC intermediate, since upregulation of NPC-associated markers was reported to be very short-lasting (Zhang et al., 2013; Busskamp et al., 2014). Likewise, transient activation of an NPC-like transcriptional program was recently described in the context of direct pericyte-to-neuron conversion (Karow et al., 2018). Interestingly, however, single cell-RNA sequencing of neural cultures derived from human PSCs via NGN2 overexpression recently indicated that even after culturing these cells in neuronal differentiation-promoting medium on mouse glia, a significant fraction of cells can remain in an NPC-like stage resisting neuronal maturation (Nehme et al., 2018). Third, in contrast to forward programming from homogenous iPSC batches, every iN represents a single, post-mitotic direct cell fate conversion event. This means that transdifferentiation-derived cultures represent a mosaic of a vast number of single conversion events, which severely limits the degree of standardization that can be reached with an iN approach. Furthermore, since neurons are post-mitotic, the cell yield of an iN conversion is always limited by (i) the number of starting cells and (ii) the efficiency of the direct conversion approach. However, these drawbacks do not equally apply to all transdifferentiation paradigms. Instead of deriving terminally differentiated iNs, direct cell fate conversion can be used to generate induced neural stem cells (iNSCs; Han et al., 2012; Ring et al., 2012; Shahbazi et al., 2016; Sheng et al., 2018) induced neuronal-like NPCs (iNPCs; Giorgetti et al., 2012), or even iNPCs which are already primed to differentiate toward a specific neuronal subtype such as dopaminergic neurons (Tian et al., 2015). As these cells are stably self-renewing, especially iNSC cultures are almost as scalable and homogeneous as PSCs. Yet, direct conversion into iNSC/iNPCs has the drawback that, as with forward programming, it has to be followed up by a subsequent terminal differentiation step. Here, it is tempting to consider translating previous approaches on facilitating the differentiation of primary or PSC-derived NPCs by TF overexpression to iNSCs/iNPCs. A key issue in all these scenarios remains cell type authenticity, which can be compromised if the converted cells retain a significant degree of epigenetic and transcriptomic memory relating to the fate but also the age of the cell of origin. This memory seems to be largely maintained in fibroblast-derived iNs of different donor ages (Mertens et al., 2015; Yang et al., 2015; Huh et al., 2016; Tang et al., 2017; Kim et al., 2018), whereas it is almost completely reset in iPSCs (Polo et al., 2010; Lo Sardo et al., 2017; Olova et al., 2019). Recently, directly converted iNSCs were shown to largely reset age-associated cellular signatures, too (Sheng et al., 2018).

**TABLE 3:** Comparative summary of key features important for cell fate engineering.

	Forward programming	Direct cell fate conversion
*Epigenetic barriers for reprogramming*	Low	High
*Scalability*	High	Limited depending on proliferative potential of converted product
*Degree of standardization that can be reached*	High	Limited depending on proliferative potential of converted product
*Preservation of somatic and age memory*	Low	Potentially high
*Possible translation into clinical applications*	Indirect (via transplantation)	Direct and indirect (via *in situ* conversion and transplantation of converted cells)

### Prospects of Direct Cell Fate Conversion *in vivo*

Direct cell fate conversion cannot only be achieved *in vitro* but also directly *in vivo*. While this concept is distinct from classic forward programming and has received its own coverage in several recent reviews (Heinrich et al., 2015; Grealish et al., 2016; Srivastava and DeWitt, 2016; Barker et al., 2018; Flitsch and Brüstle, 2019; Pesaresi et al., 2019), it might eventually provide a short-cut for brain repair bypassing a transplantation step and is thus worth mentioning here. Several studies have shown that such a transdifferentiation step can be triggered *in vivo* by the direct administration of reprogramming cues like TFs and/or miRNAs to resident rodent brain cells such as astrocytes (Buffo et al., 2005; Kronenberg et al., 2010; Guo et al., 2014; Faiz et al., 2015; Ghasemi-Kasman et al., 2015; Liu et al., 2015; Torper et al., 2015; Gascón et al., 2016) and NG2-positive glial cells (Guo et al., 2014; Heinrich et al., 2014; Torper et al., 2015; Gascón et al., 2016). In this context, the group of Ernest Arenas demonstrated that even the *in vivo* conversion of mouse astrocytes into clinically relevant neuronal subtypes such as midbrain dopaminergic neurons is feasible. The authors overexpressed a TF cocktail comprising Ascl1, Lmx1a, Neurod1, and miRNA-218 in mouse brain astrocytes by stereotactic lentivirus injection and revealed that this does not only successfully elicit transdifferentiation but finally also corrects basal and postsynaptic deficits in dopamine transmission and improves spontaneous motor behavior deficits in 6-OHDA-lesioned PD mice (Rivetti Di Val Cervo et al., 2017). Importantly, the group of Magdalena Götz recently revealed that *in vivo* conversion of mouse cortical astrocytes into neurons can preserve region-and even layer-specific identities (Mattugini et al., 2019). This study also impressively underpins the relevance and potential impact of subspecification within the glial lineage. Lastly, it has recently been shown that *in vivo* cell fate conversion can even be extended beyond germ layer boundaries: Matsuda et al. (2019) reported that brain-resident microglia, which originate from the yolk sac, are amenable to neuronal conversion in the mouse brain (Matsuda et al., 2019). A special variant of the *in vivo* conversion concept is the idea to transplant somatic cells that are already engineered to overexpress specific TFs upon an inducing stimulus and can thus be activated to convert *in situ* (Torper et al., 2013). Taken together, these reports underline the enormous biomedical potential of both, direct *in vitro* and *in vivo* conversion of somatic cells.

## Challenges for Future Clinical Application

### Cell Type Subspecification, Authenticity, and Maturity

Forward programming comes with the significant benefit of being fast enough to be able to provide autologous cells from human patients for cell replacement therapies. However, on the benchwork side, one significant limitation of some currently available forward programming protocols is the reduced purity of the obtained cultures, especially when it comes to deriving highly specified neuronal subtypes. In some cases, subspecification might be augmented by the use of morphogens and small molecules. On the one hand, these molecules can help to properly regionalize (intermediate) NPC stages, as they might provide additional phenotype-instructing differentiation cues, which could otherwise only be delivered by combining several TFs upstream of the lineage-relevant signaling pathways. This was nicely exemplified by the group of Johan Ericson and Thomas Perlmann, who showed that overexpression of Lmx1a in ESC-derived mouse NPCs suffices to instruct dopaminergic neuron differentiation when combined with Shh and Fgf8 treatment, whereas caudalized NPCs only adopt a dopaminergic phenotype if the three midbrain-associated TFs Lmx1a, En1, and Otx2 are overexpressed in combination (Panman et al., 2011). On the other hand, small molecules might act as epigenetic modifiers and facilitate forward programming by either re-activating lineage-instructive genes, which are in an unfavorable epigenetic state or even completely silenced in PSCs, or by repressing alternative lineages (similar to the mode of action that has been described for the TF EZH2; Liu et al., 2018). In direct cell fate conversion, for instance, iN derivation from human fibroblasts via NGN2 overexpression is only successful if combined with the treatment with two small molecules, namely forskolin and dorsomorphin, which modulate chromatin accessibility at NGN2 target sites (Liu et al., 2013; Smith et al., 2016).

Another challenge for the clinical application of forward programmed cells might be the degree of cellular authenticity and maturity that can be achieved. One way to improve neuronal maturation *in vitro* is to co-culture forward programmed neurons with glial cells. Notably, the species from which the glial cells are retrieved might influence their maturation-promoting effect: In a recent study, culturing human glutamatergic neurons (differentiated via a classical morphogen-based approach) on mouse astrocytes was found to be superior to co-culture with rat astrocytes, whereas co-culture with human astrocytes did not support neuronal survival beyond 4 weeks. Interestingly, GABAergic neurons did not show this selective response to glial co-culture (Rhee et al., 2019), and whether or not these effects also apply to forward programmed neurons is still to be determined.

Further studies are also required in order to clarify to what extent forward programmed neurons resemble their physiological *in vivo* counterparts. For example, in a recent study 4-week-old NGN2-forward programmed human neurons grown in an autapse setting displayed surprising morphological and functional properties, including systematic multi-peak excitatory postsynaptic currents originating from neurons possessing multiple axons, which were evident in about 25% of all cells analyzed (Meijer et al., 2019; Rhee et al., 2019). Increased axonal lengths and small diameters contributed to the observed phenomenon, and further resulted in extended synaptic delays as compared to mouse forebrain cortical neurons. Moreover, detection of a slow component contributing to these unusual electrophysiological kinetics indicated that the NGN2-neurons possess the ability to co-release GABA and glutamate from the same synapse (Rhee et al., 2019). Although there are neurons *in vivo*, which grow multiple axons and/or co-release different neurotransmitters from the same synapse, these findings deserve further attention.

### Transgene Delivery, Stability of Programmed Phenotypes and Safety

From a translational perspective, extrinsic factor-driven protocols, such as the delivery of differentiation cues by growth factors, morphogens and/or small molecules but also TF-based approaches employing mRNAs or proteins rather than integrating constructs might be easier to pass regulatory hurdles associated with the implementation of clinical trials. So far, however, most TF-based approaches have been relying on genetic modification of the target cell either by the use of integrating viruses or gene editing techniques such as CRISPR/Cas9. A main advantage of integrating a fate-instructing TF by techniques such as TALENs or CRISPR/Cas9 is better control on the integration site. Accordingly, some studies successfully integrated the forward programming-conveying TFs in the human genomic safe harbor *AAVS1* locus with high precision (Wang et al., 2017; García-León et al., 2018; Li Y. et al., 2018a; Meijer et al., 2019; Rhee et al., 2019). While integration-free methods such as mRNA (Goparaju et al., 2017; Matsushita et al., 2017; Xue et al., 2019) or protein delivery (Robinson et al., 2016) are appealing, they come with their own pros and cons. For instance, mRNAs are rapidly translated but subsequently also timely degraded after entering the target cells, and significant protocol adaptations might be necessary to enable efficient transfection at all (Xue et al., 2019). Protein delivery, on the other hand, is technically challenging but also the only technique that circumvents potential post-translational regulation (Robinson et al., 2016).

Another challenge is the maintenance of inducible transgene activation in the grafted cells until the point where the cellular phenotype of the transplanted cells becomes stable and transgene-independent. In principle, this could be tackled by grafting cells at later stages of *in vitro* specification. However, advanced pre-differentiation is typically associated with decreased survival and integration of the grafted cells. Thus, it might be beneficial to implement modalities for continuous delivery of TFs, e.g., by repetitive virus injection or slow-release depots in form of scaffolds binding or encapsulating fate-specifying proteins such as TFs and/or morphogens (see review by Bruggeman et al., 2019). Prolonged provision of fate-specifying factors beyond the timepoint of transplantation might also enhance the *in vivo* stability of neuronal subtype identities: Although there are TF-based protocols available to produce quite specific neuronal subtypes *in vitro*, the results of several studies suggest that TF-mediated acquisition and maintenance of subtype specification might be less efficient in transplanted neurons compared to a pure *in vitro* scenario (Martinat et al., 2006; Theka et al., 2013; Faedo et al., 2017; Yuan et al., 2018).

Another issue to be tackled when it comes to clinical transplantation is the fact that although grafting NPCs is more efficient than transplantation of terminally differentiated mature cells – a notion particularly relevant for neurons (for a more comprehensive commentary, see Björklund and Lindvall, 2000) – transplantation of still immature cells such as differentiating PSCs and NPCs can increase the risk of uncontrolled overgrowth (Friling et al., 2009). While growth factor-based protocols might be more vulnerable to this complication, teratoma formation after transplantation has also been observed in the context of TF overexpression paradigms (Martinat et al., 2006; Friling et al., 2009).

## Concluding Remarks

TF overexpression in NPCs and TF-based forward programming of PSCs are valuable techniques to derive specialized and comparably mature neural cells within short time frames and thus provide powerful alternatives to classic growth factor-mediated PSC differentiation and direct (somatic) cell fate conversion. Besides generating precious insights into how cell fates are established and controlled by transcriptional and epigenetic regulation, one major asset of these approaches is that they can provide new donor sources for brain repair. Yet, a number of issues need to be addressed more deeply before forward programming can be implemented in a clinical setting.

## Author Contributions

LF, KL, and OB contributed to the writing and editing of the manuscript.

## Conflict of Interest

The authors declare that the research was conducted in the absence of any commercial or financial relationships that could be construed as a potential conflict of interest.

## Abbreviations

6-OHDA: 6-hydroxydopamine
AAVS1: adeno-associated virus integration site 1
Aldh1a1: aldehyde dehydrogenase 1 family member a1
ALDH1L1: aldehyde dehydrogenase 1 family member L1
Aldh2: aldehyde dehydrogenase 2
AMPA: α-amino -3-hydroxy-5-methyl-4-isoxazolepropionic acid
Ascl1: achaete-scute homolog 1
Atoh1: atonal bHLH TF 1
ATP: adenosine triphosphate
Barhl1: BarH-like homeobox 1
Bcl-XL: B-cell lymphoma-extra large
bHLH: basic helix-loop-helix
Bmp: bone morphogenic protein
Brn2 aka POU3F2: POU domain class 3 transcription factor 2
Brn3a aka POU4F1: POU domain class 4 transcription factor 1
Brn4 aka POU3F4: POU domain class 3 transcription factor 4
ChAT: choline acetyltransferase
c-Myc: avian myelocytomatosis viral oncogene cellular homolog
CNP: 2 ′,3 ′ -cyclic nucleotide 3 ′ -phosphodiesterase
CLYBL: citrate lyase beta like
CRISPR/Cas9: clustered regularly interspaced short palindromic repeats/Cas9
Ctip2 aka Bcl11b: B-cell lymphoma/leukemia 11b
Cux1: cut like homeobox 1
Dat: dopamine transporter
Darpp32 aka Ppr1b: protein phosphatase 1 regulatory subunit 1b
Dbh: dopamine beta-hydroxylase
Dkk1: Dickkopf-related protein 1
Dlk1: delta like non-canonical Notch ligand 1
Dll: distal-less
Dlx: distal-less homeobox
Dmrt5: doublesex and mab-3-related TF 5
Ebf: early B-cell factor
Egf: epidermal growth factor
Emx2: empty spiracles-homeobox 2
En: engrailed homeobox
ESC: embryonic stem cell
Ezh2: enhancer of zeste homolog 2
Fabp7: fatty acid binding protein 7
Fgf: fibroblast growth factor
Foxa2: forkhead-box-protein 2
Foxg1: forkhead box g1
Foxo1: forkhead box o1
GABA: gamma aminobutyric acid
Gal: galanin
GALC: galactosylceramidase
GDNF: glial cell-derived neurotrophic factor
GFAP: glial fibrillary acidic protein
Girk2: G-protein -related inward-rectifier potassium channel 2
Glast: glutamate aspartate transporter
Gpx1: glutathione peroxidase 1
Gsx2: genetic-screened homeobox 2
HB9: homeobox HB9
HD: Huntington’s disease
Hes: hairy and enhancer of split
Il: interleukin
iN: induced neuron
iNPC: induced NPC
iPSC: induced pluripotent stem cell
Isl1: islet-1
Klf4: Krueppel-like factor 4
Krox20 aka Egr2: early growth response protein 2
Lhx: LIM homeobox
Lif: leukemia inhibitory factor
Lmx1: LIM homeobox TF 1
Map2: microtubule-associated protein 2
Mbp: myelin basic protein
MN: motor neuron
MSN: medium spiny neuron
Myod3: myogenic differentiation 3
Myt1l: myelin TF 1-like
Net: norepinephrine transporter
Neun: neuronal nuclei
Neurod: neurogenic differentiation
NfI: nuclear factor I
NG2: neuron-glial antigen 2
Ngn: neurogenin
Nkx2.1: homeobox protein NK-2 homolog A
Nkx2.2: homeobox protein NK-2 homolog B
Nkx6.2: homeobox protein NK-6 homolog B
NPC: neural precursor cell
Nr2f1: nuclear receptor subfamily 2 group f member 1
Ntn1: netrin 1
Nurr1: nuclear receptor related 1
O4 aka CLDN11: claudin 11
Oct3/4: octamer-binding TF 3/4
Olig: oligodendrocyte TF
OPC: oligodendrocyte progenitor cell
Otx: orthodenticle homeobox
Pax: paired box protein
PBX1: pre-B-cell leukemia TF 1
PD: Parkinson’s disease
Phox2: paired like homeobox 2
PIEZO2: Piezo-type mechanosensitive ion channel component 2
Pitx3: pituitary homeobox 3
PLP: proteolipid protein
PRC2: polycomb repressive complex 2
Prrx1: paired related homeobox 1
PSC: pluripotent stem cell
RA: retinoic acid
REST: RE1 silencing TF
Rg4 aka UNC119: retinal protein 4
Rnf20: ring finger protein 20
ROSA: reverse oriented splice acceptor
S100 β: S100 calcium-binding protein β
Satb2: special AT-rich sequence-binding protein 2
Shh: sonic hedgehog
Smad: mothers against decapentaplegic
Sox: sex determining region Y-box
Stat3: signal transducer and activator of transcription 3
TALENs: transcription activator-like effector nucleases
Tbx: T-box TF
Tcf15: TF 15
TF: transcription factor
Tfap2a: TF AP-2 alpha
Th: tyrosine hydroxylase
Tlx3: T-cell leukemia homeobox 3
TRPM8: transient receptor potential cation channel subfamily melastatin member 8
Tubb3: class III β-tubulin
Tyrp1: tyrosinase-related protein 1
VAChT: vesicular acetylcholine transporter
vGLUT1: vesicular glutamate transporter 1
VIM: vimentin
Vmat: vesicular monoamine transporter
WNT: wingless-int
Zeb: zinc finger E-box -binding homeobox
Zfp: zinc finger protein.
